# The Modelling of Extrusion Processes for Polymers—A Review

**DOI:** 10.3390/polym12061306

**Published:** 2020-06-08

**Authors:** Marko Hyvärinen, Rowshni Jabeen, Timo Kärki

**Affiliations:** Fiber Composite Laboratory, LUT University, P.O. Box 20, 53850 Lappeenranta, Finland; Jabeen.Rowshni@lut.fi (R.J.); timo.karki@lut.fi (T.K.)

**Keywords:** extrusion, screw, modelling, polymer, processing

## Abstract

Extrusion processes are widely used in industries that aim to produce advanced solutions for increasingly sophisticated demands in the plastic, food, and pharmaceutical sectors. Though the process has been in use since the 1930s, limited information is available on the analytical computation of extrusion. Generally, production has been carried out based on empirical experience and trial-and-error approaches. The development of industrial operations is, however, best addressed by modelling the processes involved, and the flow of polymer melts and fibers in extruders has been subjected to some previous studies. Also included an overview of design of a die as well as challenges in sheet/film production. This article systematically and critically reviews the literature related to the process design, machine design, process parameters, flow models, and flow analysis of extrusion with a focus on modelling the extrusion of composite materials.

## 1. Introduction

Extruders are common devices in the plastic, metal, and food processing industries, and the utilization of extrusion processes is particularly widespread in product manufacturing that uses polymers as a raw material. Typical products made from extruded polymers include, for example, pipes, hoses, insulated wires, cables, sheets and films, and tiles [1]. 

Generally, major extruders are classified as a single-screw or twin-screw type, the former being widely applied to general polymer processing and the latter for compounding various fibers, fillers, and polymer blends prior to final molding [2]. Twin-screw extruders can be further divided into two types based on the interactions of the two screws: intermeshing and non-intermeshing twin-screw extruders. In the twin-screw extruder family, fully intermeshing counter-rotating twin screw extruders have been found to have the best pumping abilities because of their positive displacement characteristics.

Intermeshing counter-rotating twin screw extruders were developed from continuous kneaders in 1939 as part of the modules intended for compounding [3], and since this time, many different extruders have been developed to improve manufacturing performance in the food, metal, pharmaceutical, and composite sectors [3,4]. In polymer-based production, extruders were initially used for the profile extrusion of pipes of polyvinyl chloride. Modular extruders, especially extruders for closely intermeshing counter-rotating twin screw extrusion, have undergone intensive experimental study [5].

Simulation is an effective way of examining, analyzing, and improving a process. Along with historical development in the design, manufacture, and application of extruders, operating conditions and parameters have been investigated analytically, and theoretical models have been presented to describe the process. This type of theoretical modeling, however, remains limited, and some parameters are still chosen on an iterative basis during manufacturing. Hence, there is a need to understand the present status of the theoretical understanding of the process, materials, and demands of the industry to enable an accurate modeling of extrusion and, possibly, aid in the development of new parameters.

In this paper, the process of extrusion is reviewed based on a part-by-part description of the process, processing technology, materials, process parameters, effects of machine elements, and structural limitations for the application of extrusion in the composite manufacturing industry. This study aims to present integrated global models to numerically simulate the extrusion process with multi-phase materials where the fill factor, pressure fields, temperature fields, and melting status are considered, as well as the feed rate under flood-fed conditions. The findings in this investigation can be used to identify areas requiring development and to improve extrusion process performance.

## 2. General Remarks

The first detailed analyses of the extrusion process were related to classical flood-fed single screw extrusion [6] and concentrated on the melt conveying process and, later, solid conveying. An early fundamental model for melting in a single screw extruder was proposed by Maddock and Tadmor et al. [6,7,8]. Melting models and various comprehensive computer models were the basis for the development for flood-fed single screw extruders, which are discussed later in the paper. However, studies on starve-fed single screw extrusion have only started very recently and mainly focus on mixing and melting capacity, with little attention given to modeling the process [6,9].

On the other hand, twin-screw extruders are commonly used in modern industry [5,10,11,12,13]. Such extruders can be divided based on the relative rotational direction of their screws into two types: co-rotating and counter-rotating twin-screw extruders. In a co-rotating twin-screw extruder, the maximum velocity is found at the screw tips, whereas in counter rotating twin-screw extruders, the maximum velocity is obtained in the intermeshing region. It can be argued that the co-rotating mechanism provides better mixing as the material is transferred between the lobes. However, the counter-rotating mechanism generates greater pressure buildup, making it more efficient for profile extrusion [14].

Many studies have experimented with different types of extruders in an effort to understand the material flow in the screws. Single- and twin-screw extruders were compared by Senanayake et al. [15] as part of a design research focused on a simplified extruder for less developed countries for extrusion cooking and to process local food materials. Single-screw extruders are simple to construct but are more likely to become clogged with material than twin-screw extruders. 

Single-screw extruder is the most common type of extruder, and it offers relatively low investment cost. Twin-screw machines are used for higher output. In extrusion, the die is a critical factor in influencing both output rate and product quality. The easiest way to increase the throughput of an extruder is to increase the screw speed. This ease solution typically results in poor melt quality caused by exceeding the melting capacity of the screw design and degradation caused by high melt temperature [16]. Use of a smaller diameter screw can offer several advantages to achieve a higher throughput at higher screw speed. One important advantage of a smaller diameter extruder is better heat transfer characteristics. A higher throughput at higher screw speed can be achieved with the use of a smaller diameter extruder. This offers better heat transfer characteristics [16].

The objective of an extrusion die is to distribute the polymer melt in the flow channel so that the material exits from the die with a uniform velocity and minimal pressure drop. Except for circular dies, it is extremely difficult to create a single flow channel geometry that can be used for a wide range of polymers and operating conditions. Through an extrusion die, the exit velocity distribution is a function of the shear-rate, temperature and the heat dissipation of the polymer melt [17]. In both single- and twin-screw extrusion, all dies require adequate and uniform heating with no dead spaces in the flow channels to prevent hot or cold spots in the polymer flow that might alter the melt viscosity or lead to resin degradation [18]. The extrusion die performance depends on the design of the manifold geometry and on the operating conditions adopted during extrusion [17]. The design of extrusion die is a complex task because the extrudate product dimensions depend on the die design and on the polymer properties and extrusion process parameters. The die design process can be improved by integrating computational simulation with empirical data and by improving the extrusion monitoring instrumentation. A better and developed die design improves product quality and reduces time to design and optimize the extrusion process, and thus results in lower costs. [19] 

In twin-screw extruders, since the flight of one screw engages with the channel of the other, blockages can be prevented, thereby enhancing the mixing of material in the channels of the screws. In single-screw extruders, the material is retained for much longer than in twin-screw extruders, a phenomenon associated with stagnant layers on the screw surface [20]. A twin-screw extruder has about three times more material output than a single-screw extruder of a similar size and screw speed [21]. 

Theoretically, the material flow process can be divided into four sections: (a) feeding of the extruder, (b) transport of mass, (c) flow through the die, and (d) exit from the die and subsequent downstream processing. During material processing, the mass is transformed mainly by the shear force, pressure, cooling rate, shaping, and residence time [22]. Conventionally, the extrusion channel is divided into three parts: (a) the feed zone, (b) the transition zone, and (c) the metering zone. The material processing time in the extruder is termed the residence distribution time.

Residence time distribution is an important parameter for product quality. The residence time distribution in intermeshing counter-rotating twin-screw extruders has been studied widely [20,23,24,25,26]. The study of Sakai et al. [20] compared the residence time distribution in counter-rotation and co-rotation twin screw extruders and found that a sharper residence time distribution was obtained when using the counter-rotating twin screw extruder. Janssen et al. [23] studied residence time distribution by injecting a pulse tracer containing radioactive magnesium oxide into polypropylene into a Pasquetti twin screw extruder. The reproducibility of the results of the decayed magnesium (Mn) and ferrum (F) was detected using a scintillation crystal. This was used to determine the exit age distribution [21]. This work was followed by that of Wolf et al. [25], in which a radioactive magnesium oxide was applied to a Krauss–Maffei Model KMD 90 machine that was used to extrude polyvinyl chloride. Shon et al. [26] compared the residence time distribution in four types of continuous mixers and concluded that the intermeshing counterrotating twin-screw extruders had a narrower residence time distribution than the modular co-rotating twin screw extruders and kneaders. This finding agrees with the work of other authors, who found that the residence time distribution is narrower for intermeshing extruders than for single-screw extruders or co-rotating twin-screw extruders [21].

Most commercial extruders provide a choice of screws or interchangeable sections that alter the configuration of the feed, transition, and metering zones. This kind of modular design makes it possible to modify the extrusion process to meet specific requirements, such as mixing [1]. 

Starve-fed screws are mostly seen in twin-screw extruders. The throughput of the extruder is not a function of the screw speed at a steady state. Unlike melting in single-screw extruders, studies of melting in twin screw extruders have only appeared recently in the literature [12]. Various models have been developed to analyze the melting process in twin screw extruders. Based on these melting models, several comprehensive computer models have been developed, mostly for co-rotating twin screw extruders [9].

## 3. Process Modelling

In order to model a process, it is important to choose the right simulation tool to analyze the operation practically and realistically. For polymer composite extrusion, the simulation tool should be equipped to handle aspects essential to the process. For example, conditions in the barrel are not isothermal and isobaric [27], and, in most cases, the material is not homogeneous. This lack of homogeneity is further complicated if the process involves granules [28] or fibers [29,30]. In 1990s, non-isothermal flows were studied by Kye et al. [31] and White and Chen [32,33] in co-rotating twin screw extruders, by Bang and White [34] in tangential counter-rotating twin screw machines, and by Hong and White [35] for intermeshing counter-rotating twin screw extruder.

The flow of polymers in the extrusion process has been well defined, although it remains difficult to model the flow in twin-screw extruders. More advanced modelling requires a composite approach that includes scrutiny of the solid conveyance, melting, and melt flow of the polymer. The first detailed analyses of the polymer extrusion process were related to melt conveyance and, later, solid conveyance. Based on these models, various comprehensive computer models for the extrusion processes were developed. As most twin-screw extruders are self-wiping and starve-fed [36], more models have appeared for such extruders in recent studies [9,37].

The flow of a molten polymer can be represented in a global model using 1D and 2D approaches [6,8,37,38]. This work involves simplifying a particle path and performing a finite volume analysis, which requires knowledge of the screw geometry, material properties, and processing conditions. Common extrusion modeling tools for such analyses include LUDOVIC^®^, Morex, SIGMA, and Akron-Co-Twin Screw^®^ [39,40,41]. The main process parameters are pressure, temperature, residence time, and filling ratio.

LUDOVIC^®^ is simulation software for macro thermo–mechanical behavior in twin-screw extrusion and batch processes. The approach is 1D non-isothermal along the screw length and allows the user to calculate the evolution of the main process parameters. In an ultra-high speed extrusion study [42], for example, the LUDOVIC^®^ software was used to calculate the thermomechanical flow parameters for various screw profiles and was found to offer a good correlation between the theoretical and experimental results.

Wilczynski et al. [9] used the simulation system TWIN_CT (i.e., a counter rotating twin-screw extrusion model) for various screw configurations. This model presents the three main areas of the process: solid conveyance, melting, and melt flow. The simulation makes it possible to predict the course of polymer melting, pressure extrusion, and temperature profiles, as well as the degree of screw channel filling in a counter rotating twin screw extruder [41]. This model is very efficient for a modular screw arrangement. A technique based on 3D FEM modeling was presented by Ishikawa et al. [43] as a numerical model for a co-rotating twin screw extruder. This model claims to be a powerful tool for mixing performance evaluation because it enables the estimation of mixing parameters such as the RTD and stress and strain history.

The melting model by Wilczynki and White [37] describes the pressure effects in the calender gap of twin screws on the melting of polymers. The polymer melts and flows out from the calendering gap—the pressurized end of the C-chamber—in a pressure flow. This model provides a theoretical justification for the use of counter-rotating screws. Wilczynki et al. [9] generalized the approach for plastic process modelling for single-screw equipment, and Baronsky-Probst et al. [10] did the same for twin-screw extrusion.

In most cases, approaches are similar with slight variations when there is a change in the process conditions, as demonstrated by Redl et al. [39] for self-swiping and starve-fed extruders. The filling ratio was unknown for the system, so the computation was done in a backwards direction from die to feed. Because of the unknown temperature of the final product, an iterative procedure was used to calculate the pressure, fill factor, and temperature profiles. The calculated temperature at the position where the melting finishes is compared to the melting point of the plastic used. The convergence of the iterative computations is obtained when the temperatures are equal [5,9].

In a numerical analysis of the flow of polymers in rotating screws, a material is considered to be in a liquid form from the feeding zone [36]. On the other hand, when melting is observed experimentally, it is influenced by factors like feed rate, screw speed, and shear rate, indicating that melting is a gradual process. Experiments conducted by Lewandowski et al. [5] show that melting begins earlier when the feed rate increases because of the faster formation of a pallet bed. When the C-chambers are more completely filled, and the length of the pallet bed (a layer formed by semi or fully melted polymer in the barrel) is longer, the complete melting of the polymer requires longer screws. When the screw speed at a constant flow rate is increased, melting takes more time since the polymer pallets are transported faster, and the degree of fill decreases [44]. However, since the C-chamber is less filled, shorter screws are needed for complete melting. The model by Wilczynski et al. [45] distinguishes two regions of melting: a partially filled region of melting and a fully filled region of melting. Based on the melting phenomena, mathematical models were proposed for melting in both regions [5,6]. In the partially filled region, an energy balance was applied on an elementary volume of the material under the assumption that there is no friction heat generated in this region.

About 80% of the heat required to melt or fuse the mass is supplied by the heat generated from the friction between the screws and the barrel, resulting in shear. The remaining heat is supplied by external sources, such as sets of heat cartridge heaters positioned in grooves in the barrel [14,46,47]. 

Most extrusion process models have simplified modelling by neglecting some parameters. The extrusion process was optimized by Malik et al. [48] through the inclusion of wall-slip conditions at the barrel and screw surfaces. In mixing zones consisting of combinations of forward- and reverse-conveying screw elements, wall slip decreases the pressure increase rate of the forward-conveying screw elements and the pressure loss rate of the reverse-conveying screw elements, yielding an overall decrease of pressure in the mixing section. This suggests that the process can be optimized by controlling the wall slip behavior of the fluid via a judicious selection of materials, surface roughness, and temperatures for the screw and barrel surfaces of the extruder and the die. 

**Figure 1:**
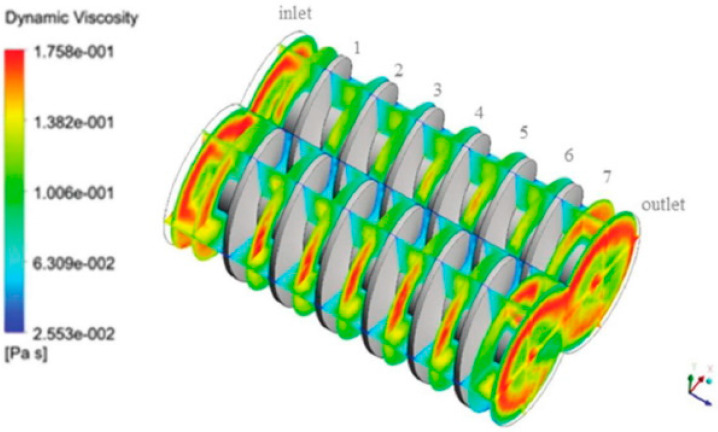
Computational fluid model viscosity contour of twin-screw extruder’s feeding zone with ANSYS software adapted from Tagliavini et al. [49] with permission from De Gruyter, 2020.

Most recently, Polyflow, a finite element CFD software developed by ANSYS, was used to simulate extrusion processes of both single- and twin-screw extruders [5]. The temperature field, pressure variation along the screw, flow pattern, residence time, and shear forces were predicted. Lewandowski et al. [5] and Lewandowski [12] applied an approach using fully three-dimensional non-Newtonian FEM modelling to describe the screw pumping characteristics in an intermeshing counter-rotating twin-screw extruder with polymers. The results were validated experimentally. The non-Newtonian shear thinning behavior of molten polymers is usually modelled by the power law model or the logarithmic Klien equation. However, the pressure profiles for a Newtonian and a non-Newtonian fluid are generally similar, and the pressure gradient decreases with a decrease in the power law index [5,12]. An example of fluid simulation is the study of Tagliavini et al. [49] were ANSYS FLUENT was used to simulate a computational fluid dynamic model of a twin-screw extruder’s feeding zone. Figure 1 shows the viscosity contour of the screw section at the feeding zone, one of the simulated results.

**Figure 2:**
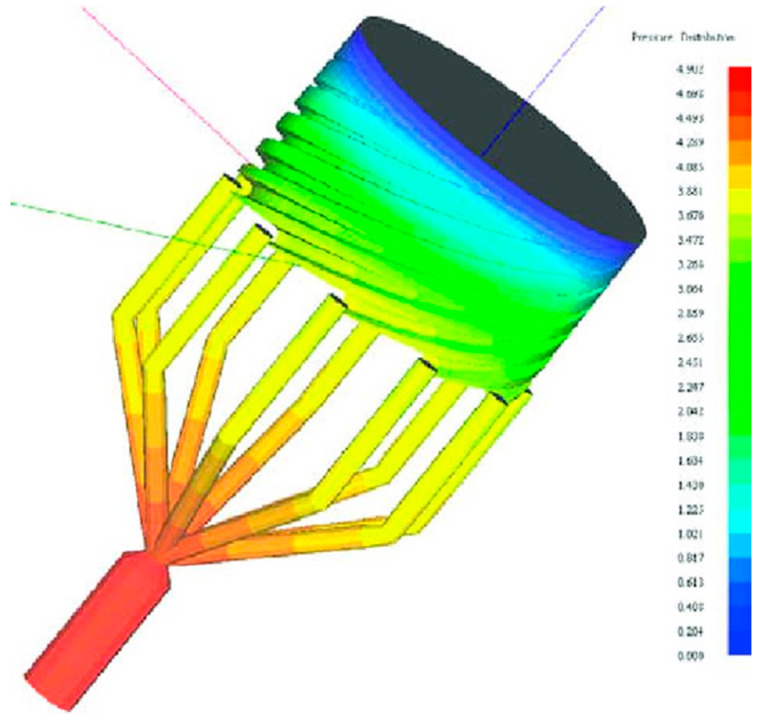
Pressure distribution predicted by SPIRALCAD ADVANCE in modelling of polymer film blowing adapted from Vlachopoulus and Sidiropoulos [52] with permission from Elsevier, 2020.

Because of the rapid increase of the new applications of film/sheet products in various fields of packaging, electronic, and optical industries, the higher productivity with lower manufacturing and the higher functionality are the major directions for the development of film/sheet extrusion technology [2]. Process modeling has evolved from the first mathematical and physical models made by Pearson and Petrie [50,51] to the use of modern modelling platform. For example, Vlachopoulus and Sidiropoulos [52] utilized SPIRALCAD ADVANCE software to simulate the spiral die design for blown film extrusion (Figure 2).

## 4. Process in the Barrel

The feed throat of the extruder introduces the material to the screw channel. The throat usually fits around the first few flights of the extruder screws. To prevent an early increase of the temperature in the feed throat area, the casing is generally water-cooled. At extremely high temperatures, the polymer may stick to the surface of the feed opening, causing a restriction of the flow into the extruder and leading to solid conveyance problems [24]. To provide a steady flow through the hopper, the gradual compression in the converging region should be considered, and the cross section of the hopper should be circular. 

Modern extruders have a modular screw design to facilitate efficient mixing in the barrel. Thick flight designs, thin flight designs, and mixed thick/thin flight designs have been used [15,36,39] to construct screws because the shear conditions are controlled with the flight and the rotational speed. Wilczyński et al. [9] presented a state-of-the-art technique for comprehensive modeling of screw processing of plastics. The multipurpose computer system studied the transport, melting, and mixing of material and pressure generation to push materials through the die of an extruder. Optimization procedures of the model based on the genetic algorithms, that imitate the natural evolution process, and the response surface were given by the mathematical models of the process. The study emphasized the importance of prediction of material behavior, such as melting properties and thermomechanical history, during the screw processing.

In the barrel polymeric material is affected by temperature and screw speed, but also by time. In extrusion, a term residence time distribution (RTD) is used to describe the distribution of time that polymeric material stays inside the barrel and the die. [53]. The residence time distribution (RTD) and pressure around the die have a direct effect on the profile of the product. RTD has a major influence on the product characteristics as it defines the time of the material’s exposure to temperature, pressure, mixing, and shearing geometries. The influence of RTD can be traced with near infrared (NIR) spectroscopy and the use of a UV-absorber. For example, with pure polypropylene, the residence time distribution can be measured by determining the wave number and intensity of a specific UV-absorber peak at 6475 cm^−1^ [11]. The RTD is influenced by the mean residence time (MRT), which in turn is affected by the configuration of the screw. For a twin-screw extruder, Gautam and Choudhury [54] observed that the type, length, and position of the mixing elements, as well as the spacing between the two elements, affect the MRT significantly. The residence time of the mass in the extruder is considerably increased when mixing elements are included in the screw profile. The residence time was doubled by the inclusion of reverse screw elements. Also, it was found that if the position of the mixing elements was moved away from the die, the MRT increased. Also, the increased spacing between the mixing elements increased the MRT with the growth in the length of the elements. The other earlier studies [55,56,57,58,59] showed that the MRT decreases by increasing the feed flow rate and screw speed and by decreasing the feed’s moisture content.

Mixing elements are commonly used in extruders. The kneading block and its individual disks comprise the dominant dispersive mixing unit of a fluid system. Unlike the conveying element, the mixing element generally operates when fully filled with material and may be partially or fully dependent on the pressure-driven flow [28]. The available volume of the material flow is only affected by the disk thickness. Thompson et al. [28] used mixing elements to explain the effects of the screw configuration on wet granulation. In addition to kneading disks, they used comb mixing elements to allow the splitting and recombining of flow streams with different shear histories. Using forwarding comb mixers created a more robust mixing region in this experiment. The motion of modular rotors caused pressure fluctuations as observed by Bravo et al. [60].

There are two main types of die holding structures for extruders—straight-through systems and crossheads. In the former, the die is held in line with the extruder screw, and in the latter, the extrudate emerges at right angles to the screw. Crosshead systems are capable of applying rubber compounds to a substrate, which can be a continuous material, such as a wire or cable, or a discontinuous material, such as a roller center or a mandrel [61].

Die structure has been analyzed and optimized using finite element methods. Such research has generally focused on metal extrusion dies because of the high pressure and temperature involved [62,63]. Throughput increases linearly with screw speed. The specific output seems to be independent of pressure over a wide range of melt temperatures and screw speeds.

**Figure 3:**
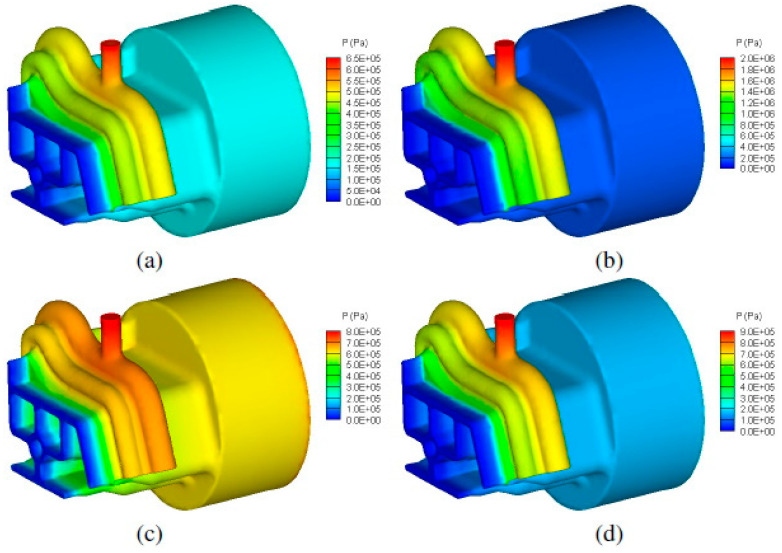
Modelling of pressure distribution in the profile die with ANSYS polyXtrue software by adapted from Gupta [65] with permission from Gupta, 2020. (**a**) Substrate: LDPE-A, Cap: LDPE-A, (**b**) Substrate: LDPE-A, Cap: LDPE-D, (**c**) Substrate: LDPE-D, Cap: LDPE-A, (**d**) Substrate: LDPE-A, Cap: LDPE-A with larger flow rate (LFR) through the cap layer.

The emergence of new demanding applications in the plastic industry has led to die profiles of increasing complexity being utilized for production, which naturally promotes unbalanced flow [61]. A rapidly growing area of research is multilayer films, which are increasingly used in packaging to achieve specific performance requirements. New polymers and processing technologies have strengthened the development of multilayer films [64]. Coextrusion is a common method used for producing multilayer blown films. In general, several measures can be taken to eliminate the non-uniform flow of material through the die, such as changes to the shape of the die, porthole location, porthole size, and local bearing length. In practice, uniform flow is mandatory for good product quality, especially during profile extrusion. In modelling of a uniform flow, process and die designers, process operators, and die correctors have switched from trial-and-error methods [63] to utilize modern methods such as computational fluid dynamics (CFD) simulation [49]. In the case of coextrusion, the velocity and stresses are required to be continuous across the interface between the adjacent layers of the multilayer flow of polymers [64]. The effect of polymer viscosity on the interface shape, velocity, pressure, shear rate, and residence time distribution. With using the ANSYS polyXtrue software, Gupta [65] found that the viscosities of the two polymers had a significant effect on the interface shape, velocity, pressure, and shear rate in the die but only a minor effect on the residence time distributions of the two polymers. Modelling of pressure distribution in the profile die for three different combinations of LDPE polymer in the substrate and the cap layer shown in Figure 3.

## 5. Reinforcements in Processing

The increasing cost of pure polymer materials has resulted in the need for less expensive reinforcement or filler materials that do not negatively affect the strength and wear resistance of the resulting polymer profile. One such filler material is wood in the form of both flour and fibers. In addition, the increasing cost of timber and timber frames in construction products means that there is a demand for alternative solid products. Accordingly, the use of plastic extrusion profiles as a substitute for wood products in door and wall applications, as well as window frames and molding, has increased in recent years. The use of cheaper polymer as a filler material, for example in production of polymer films, is a significant way of reducing raw material costs. Also, the interest in the use of recycled polymers has also increased remarkedly because of tightening legislation. The replacement of virgin polymers with recycled ones offers an opportunity to reduce raw material costs in those application where the use of recycled polymers is allowed. Growing interest has been focused in the use of other natural materials, such as hemp and sisal, as reinforcements for thermoplastics. Cellulose or cellulose-based fibers require coupling agents to improve their adhesion with matrices. Maleic anhydride-grafted polyolefins, isocyanates, and silanes are commonly used coupling agents that improve not only the adhesion but also the mechanical properties of a composite [66]. Polymer composites reinforced with wood or other natural fiber materials have shown remarkable improvements in the physical properties of polymeric materials.

Experiments have been done using new natural fibers, such as sunflower hull sanding dust (SHSD), which has been used as a composite with polypropylene [29]. Although obtaining the optimal processing conditions for extrusion has been iterative, some observations can be made about the fiber behavior in the extruder screws. The addition of SHSD influenced the crystallization behavior of the composite. Additionally, the crystallization temperature of polypropylene increased, while the melting temperature remained constant. Crystallization of polymers affects, for example, thermal, mechanical, and chemical properties of the polymer. Crystallization or flow-induced crystallization as polymer properties are not discussed in this review article.

The moisture absorption of the wood is one difficulty presented by the addition of wood flour. The loss of moisture within the extrudate can slow the extrusion rate as a result of an increase in viscosity [4]. Moreover, unlike pure polymers, the addition of heat to wood flour does not improve the flow ability of the extrudate. Encapsulating of the wood fibers with resin and palletizing them can be used to improve processability during extrusion where these palletized pieces can be mixed with additional resin and other process agents. Though this process requires double extrusion, it provides better wetting and smoother skin in the final product [4]. Incorporating the proper amount of reinforced fibers (5–20 vol %) into thermoplastic resin significantly improves dimensional stability, tensile strength, modulus, electrical properties, and corrosion resistance [30]. These properties are related to fiber concentration, length, diameter, and distribution. To increase composite strength, the average fiber length in a matrix should exceed the critical length as much as possible without interfering with processability [30]. The extrusion conditions are directly related to the mechanical properties of the product. Although work has been carried out to experiment with parameter evolution, it is sensible to simulate the process for cost-efficient experiments with the ability to change process parameters, thus leading to extrusion optimization.

## 6. Process Effects on Fibers

Over the past decades, many authors (e.g., [67,68,69,70]) have investigated the effect of fiber damage on properties of composites during compounding and extrusion. Different process factors such as screw geometry and speed, fiber dimensions, feed rate, barrel temperature, and polymer viscosity affect fiber breakage in the screws. Some studies [71,72,73] have tried to model the fiber damages in screws but modelling was also found to be challenging. For example, Berzin et al. [73] pointed out that it is important to merge modelling software and evolution laws for fiber dimensions. The reduction in fiber length is most severe during the first processing stage when the fiber bundles are being filamentized. Albrecht et al. [72] noted that the splitting of fiber bundle has to be implemented in simulation to improve the reliability of the model. 

There is a limitation to the fiber length in the composite extrusion process. To increase the composite strength, the average fiber length in a matrix should exceed the critical length as much as possible [30]. Therefore, it is important to evaluate the effect of processing variables on the extent of fiber degradation during extrusion. Reductions in fiber length and distribution have a negative structural impact on the composite product. A shear stress concentration occurs near the fiber ends, which is where failure initiates. Greater fiber breakage leads to more fiber ends, which act as sites for stress concentration where the initiation and propagation of interfacial cracks occur, thereby leading to tensile stress failure [30,74].

**Figure 4:**
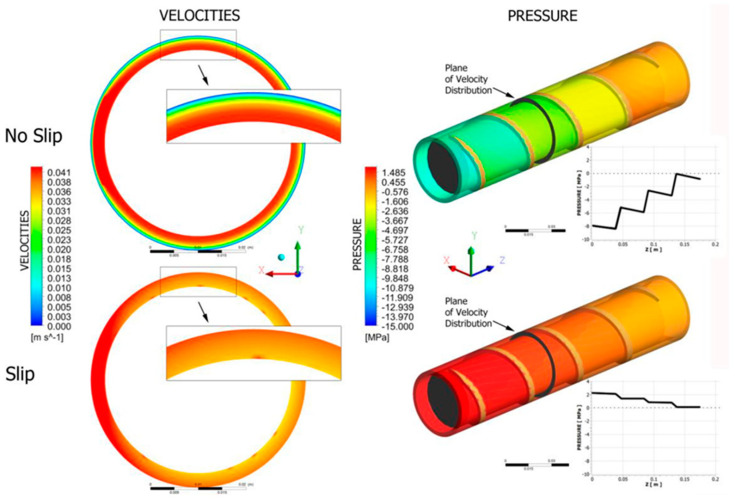
Screw flow simulations of single screw extruder adapted from Wilczyński et al. [76].

Several studies [30,46,75] have performed experiments and chosen parameters based on existing studies and iterative methods. It is challenging to experiment with new materials and material combinations because there are limitations on the extruder’s conditions, especially when working with fibers, making numerical analysis very difficult. The pressure in a twin-screw extruder can be controlled with reverse conveying (i.e., a pressure release) and forward conveying (i.e., a pressure increase) in the screw sections. The model by Wilczyński et al. [9] clearly shows that pressure is generated in the fully filled regions of the screws only—that is, at the end of the screws close to the die in the region of the shearing elements. Generally, the pumping capacity of the screws decreases if the fluid being pumped is non-Newtonian and increases as the fluid becomes more Newtonian [9]. Wilczyński et al. [76] modelled slip effects in single-screw extrusion of wood–plastics composites. Screw flow simulations, shown in Figure 4, revealed that the velocity profile drastically changed, and pressure substantially dropped in the extruder barrel and the die. The slip at the screw and at the die have an important impact on the flow rate and extrusion pressure. They found that increased slip at the screw decreases both the flow rate and pressure, while increased slip at the die increases the flow rate and decreases the pressure.

Fiber degradation under low shear deformation is negligible (as presented in the study of Hausnerova et al. [30]) based on a comparison of the fiber length distribution before and after extrusion. As mentioned earlier, extrusion with fibers is efficient when using multiple extrusion and palletizing. The apparent viscosity at a constant shear rate as a function of the number of extrusions decreases gradually as extrusions are repeated because of the degradation of the matrix polymer and a reduction of the fiber length. An increase in the number of extrusion cycles decreases the length of the fibers. During the first extrusion, severe damage to the fibers was already observed regardless of the extrusion speed and shear rate used [30].

## 7. Conclusions

The work done in past years on extrusion has been explored in this article. Though the extrusion process is well-known and commonly used in the manufacturing industry, advancements in materials are leading to new requirements. Many authors have investigated the mechanisms of the melting, mixing, and metering of polymers. There is, however, limited literature on the same functions with multi-phase materials. For mechanical product extrusion, structural property optimization is the final objective, and the key to determining the optimal process parameters is iteration. Finding relationships between product properties and process parameters is complicated, expensive, and limited if experimental data alone are used. Successful modeling and simulation can thus enable rapid and cost-effective development.

Research on fiber breakage because of the shear forces exerted through the molten matrix has been of special interest as fiber breakage is directly associated with the structural properties of the final product. There is a need to further investigate the factors causing this breakage to enable optimization of the process, as well as a need to examine the evolution of process parameters like pressure, flow rate, and temperature to facilitate effective control of the process. Simulation tools or finite element analysis models, as discussed in the paper, have historically concentrated only on pure polymer extrusion in many cases. Information on materials containing particles is limited. A simulation tool is required to enable the modification of screw characterization, analysis of the effects of the addition of screw elements, determination of fiber materials, and accurate extraction of the parameter profiles.

Multiple extrusion is a common practice used to improve the mixing of fibers and polymers. It is possible to design an extrusion screw structure to avoid multiple extrusion and reduce fiber degradation. Although lower shear stress leads to low fiber degradation, which is neglected in the literature [30], the apparent viscosity at a constant shear rate decreases with the number of extrusions due to the degradation of the polymer. The question remains if degradation can be enacted with simulation tools and if changes in the properties of the final product can be observed effectively.

Extrusion is an energy intensive process. Therefore, the optimization of process energy usage while maintaining polymer melt stability is essential to produce high product quality at low unit cost. Thermal stability and energy efficiency are strongly influenced by process conditions; polymer material and extruder used as well as process control and monitoring system [77]. The process engineering and modelling plays a key role in improving the energy efficiency of extrusion process. Screw assemblies and extruder dies are two major design areas that have a significant impact on the degradation of particles and the mixing of materials. A modifiable design could be created to analyze screw efficiency, and the design could be computationally optimized before proceeding to manufacturing in an extruder machine. There are many different software platforms, such as SOLIDWORKS and ANSYS, available on the market that include packages to design machines as well as to analyze or simulate processes. This allows easier data transfer between different packages which shortens design time and improves productivity. 

An optimized length of fibers is needed to ensure a good product. In some cases, the critical length has already been defined (e.g., [30]), and these data can be used as a reference to model the extrusion process with fibers. In the major break-up region, three possible breakage mechanisms occur: fiber–fiber, fiber–barrel, and fiber–polymer breakage [9]. This breakage is directly related to screw design, leading to a need to optimize the screw configuration and screw speed. The research done to date was carried out in polymer efficient extruders, as there was a limitation on machine availability.

As noted in this paper, there are already several extrusion modelling tools in the market. Most of the software has been designed for polymer (fluid) extrusion. Although multi-phase extrusion is possible in some cases, the approach faces limitations when fiber composites are used. The development of a universal extrusion modelling tool would facilitate the more efficient and widespread use of extrusion in modern manufacturing.
